# Indigenous microbiota protects development of medication-related osteonecrosis induced by periapical disease in mice

**DOI:** 10.1038/s41368-022-00166-4

**Published:** 2022-03-21

**Authors:** Wen Du, Mengyu Yang, Terresa Kim, Sol Kim, Drake W. Williams, Maryam Esmaeili, Christine Hong, Ki-Hyuk Shin, Mo K. Kang, No-Hee Park, Reuben H. Kim

**Affiliations:** 1 State Key Laboratory of Oral Diseases & National Clinical Research Center for Oral Diseases & Department of Prosthodontics, West China Hospital of Stomatology, Sichuan University, Chengdu, China; 2 The Shapiro Family Laboratory of Viral Oncology and Aging Research, Los Angeles, USA; 3 Section of Restorative Dentistry, UCLA School of Dentistry, Los Angeles, USA; 4 Department of Orofacial Sciences, UCSF School of Dentistry, San Francisco, USA; 5 UCLA Jonsson Comprehensive Cancer Center, Los Angeles, USA; 6 David Geffen School of Medicine at UCLA, Los Angeles, USA

**Keywords:** Experimental models of disease, Bone

## Abstract

Bacterial infection is a common finding in patients, who develop medication-related osteonecrosis of the jaw (MRONJ) by the long-term and/or high-dose use of anti-resorptive agents such as bisphosphonate (BPs). However, pathological role of bacteria in MRONJ development at the early stage remains controversial. Here, we demonstrated that commensal microbiota protects against MRONJ development in the pulp-exposed periapical periodontitis mouse model. C57/BL6 female mice were treated with intragastric broad-spectrum antibiotics for 1 week. Zoledronic acid (ZOL) through intravenous injection and antibiotics in drinking water were administered for throughout the experiment. Pulp was exposed on the left maxillary first molar, then the mice were left for 5 weeks after which bilateral maxillary first molar was extracted and mice were left for additional 3 weeks to heal. All mice were harvested, and cecum, maxilla, and femurs were collected. ONJ development was assessed using μCT and histologic analyses. When antibiotic was treated in mice, these mice had no weight changes, but developed significantly enlarged ceca compared to the control group (CTL mice). Periapical bone resorption prior to the tooth extraction was similarly prevented when treated with antibiotics, which was confirmed by decreased osteoclasts and inflammation. ZOL treatment with pulp exposure significantly increased bone necrosis as determined by empty lacunae and necrotic bone amount. Furthermore, antibiotics treatment could further exacerbate bone necrosis, with increased osteoclast number. Our findings suggest that the commensal microbiome may play protective role, rather than pathological role, in the early stages of MRONJ development.

## Introduction

Anti-resorptive treatment (ART) with agents such as bisphosphonate (BPs) or denosumab (Dmab) is commonly practiced in patients with osteoporosis and bone metastatic diseases.^1^ In patients undergoing a long-term ART, a rare intraoral lesion called medication-related osteonecrosis of the jaw (MRONJ) may develop, which is clinically defined as exposed bone through intraoral and/or extraoral fistula that persists for more than 8 weeks without a history of radiation therapy.^2,3^

Several risk factors are known to be associated with MRONJ development such as duration and/or administration route of medication,^4,5^ dentoalveolar surgery,^6–8^ and local inflammatory lesion.^9,10^ Among them, dentoalveolar trauma is one of the major risk factors for development of MRONJ. It has been reported that a tooth extraction or dental surgery is the precipitating event that causes ONJ in 78% of the cases.^2^ Studies also showed that extraction can increase the risk of having ONJ by 33 folds.^3,6^ Given that most tooth extractions are operated to resolve pre-existing pathological lesions including periodontal or periapical diseases,^3,11,12^ it has been suggested that the pre-existing local inflammatory lesions initiated by bacterial infection may already have predisposed the affected area to developing MRONJ following tooth extraction.^13–17^

Bacterial infection and host’s inflammation play an important role in development of periodontal and periapical diseases.^18,19^ Studies have shown that pathogenic bacteria colonize tooth extraction sites in animals and humans with bisphosphonate treatment, exacerbating ONJ development.^20–22^ In line with this observation, cancer patients exhibit a decrease in ONJ incidence upon improved oral hygiene, which indicates an important role of bacterial infections in MRONJ.^23^ Microbial biofilm formation on sequestered bone has been revealed by using scanning electron microscope analysis,^24^ and fluorescence in situ hybridization using the probe targeting 16s rDNA showed the presence of increased bacterial staining in ONJ lesions in an animal model.^25^ Collectively, these studies suggest that the presence of bacteria is associated with MRONJ; however, its etiological role in initiating MRONJ development is still elusive.

We recently attempted to address this question by using a mouse model that combines ligature-induced periodontitis and extraction-induced MRONJ under a condition that significantly suppressed bacterial loads with wide spectrum antibiotics (ABX). Surprisingly, ABX treatment further exacerbates the extraction-induced MRONJ with the pre-existing periodontitis, suggesting that indigenous microbiota protects from developing ONJ.^25^

Unlike the periodontal disease, periapical diseases result from the penetration of bacteria through the infected pulp, with subsequent inflammation and bone resorption at the root apex of affected tooth. Therefore, we deleted bacterial loads using broad spectrum ABX and assessed bone mass, osteonecrosis development, osteoclast, and immune cell number, trying to investigate whether indigenous bacteria function as an etiological factor in initiating MRONJ using our previously established mouse model that combines pulp-exposed periapical periodontitis and tooth extraction-induced MRONJ.^26,27^

## Results

### Broad-spectrum antibiotic treatment prevented pulp exposure-induced periapical radiolucency in mice

**Fig. 1:**
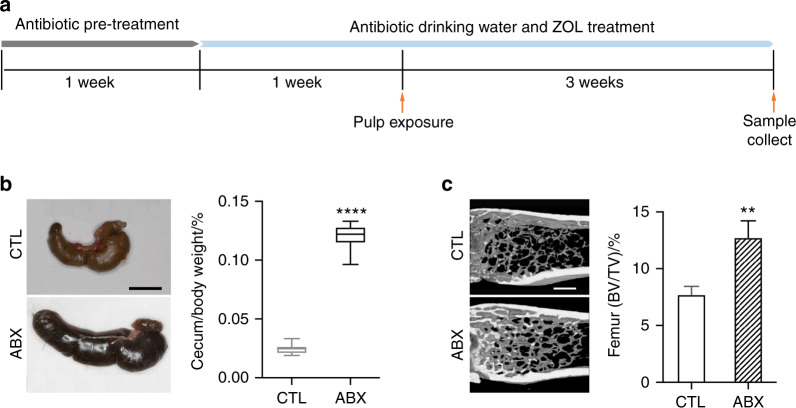
Broad-spectrum antibiotic treatment alters cecal weight and bone volume. **a** Schematic of pulp exposure mouse model with broad-spectrum antibiotic treatment. For a detailed description, please refer to section of “Materials and methods”. **b** Representative images of ceca from control (CTL) and antibiotic treated (ABX) mice and the percentage of cecum weight to total animal body weight at the time of sacrifice. **c** Representative images of the metaphyseal trabecular bone from the distal femur and quantification of bone volume (BV/TV). All quantified data represent mean ± SEM (*n* = 8). **P < 0.01; ****P < 0.000 1. The scale bars represent 10 mm in **b** and 500 μm in **c**

**Fig. 2:**
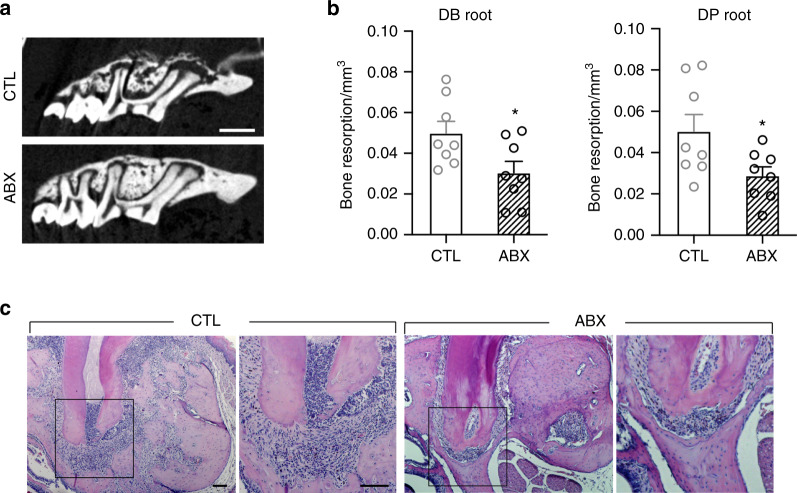
Antibiotic treatment prevents the development of periapical radiolucency (PARL) and inflammation at the apex. **a** µCT scans of pulp exposed tooth from CTL and ABX mice. **b** Quantification of total bone resorption in the apex of distobuccal (DB) and distopalatal (DP) roots on the exposed first maxillary molar. **c** Representative images of H&E staining at the apex of pulp exposed tooth. All quantified data represent mean ± SEM (*n* = 8). *P < 0.05. The scale bars represent 500 μm in **a** and 100 μm in **c**

We first aim to examine the effect of reduced commensal microbiota on apical periodontitis after pulp exposure. To do so, a mixture of intragastric and intraoral gavage of antibiotics (ABX) or PBS were administered for 1 week. Then, antibiotics were administered in the drinking water for additional week, after which the pulp was exposed. Mice were left for 3 weeks after pulp exposure until sacrifice (Fig. 1a). Consistent with the previous findings,^25^ ABX-treated mice exhibited enlarged ceca (Fig. 1b) and increased bone volume (Fig. 1c), both of which are important physiologic phenotypes associated with antibiotic-treated mice due to depleted oral and GI microbiome.^28^ When bone resorption around the root apexes were examined, ABX-treated mice exhibited less bone loss compared to the saline-treated mice (Fig. 2a, b), suggesting that broad-spectrum antibiotic treatment reduced commensal microbiota and prevented bone resorption at the root apexes mediated by pulp exposure.

### Broad-spectrum antibiotic treatment reduced numbers of osteoclasts and inflammatory cells at the apexes of pulp-exposed tooth in mice

**Fig. 3:**
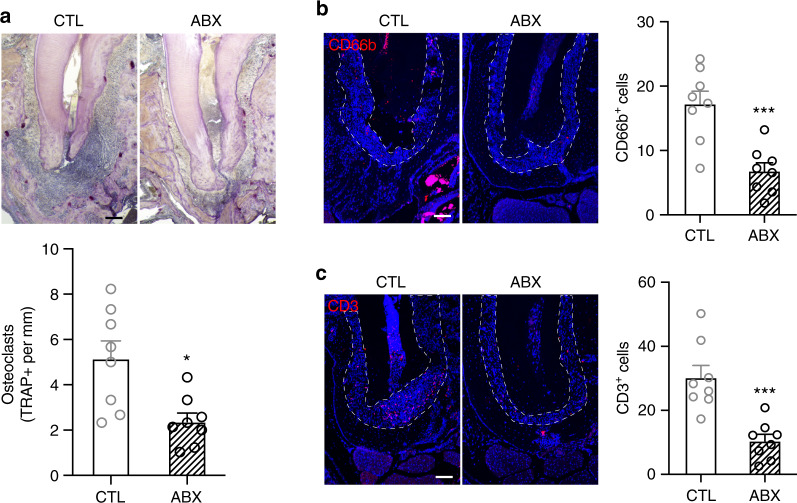
Antibiotic treatment reduces the number of osteoclasts and immune cells at the apex area of pulp exposed tooth. **a** Representative images and quantification of TRAP + osteoclast number at the apex of pulp exposed tooth. **b**, **c** Representative images and quantification of immunofluorescent staining for CD66b^+^ (**b**) and CD3^+^ (**c**) cells at the apex area of pulp exposed tooth. Dashed lines outline the area where CD66b^+^ and CD3^+^ cells were counted. All quantified data represent mean ± SEM (*n* = 8). *P < 0.05; ***P < 0.001. Scale bar: 100 μm

To further examine the effect of reduced commensal microbiota on apical periodontitis after pulp exposure at the cellular level, we assessed the presence of osteoclasts and inflammatory cells. Histological examination revealed intense infiltration of inflammatory cells at the apex of saline-treated mice. With ABX treatment, the inflammation at the apex was diminished (Fig. 2c). Similarly, the numbers of TRAP + osteoclasts were decreased (Fig. 3a). In line with these observations, we detected less numbers of CD66b^+^ granulocytes and CD3^+^ T-cells (Fig. 3b, c). These data indicate that ABX prevented pulp exposure-induced bone resorption and periapical radiolucency by reducing the numbers of osteoclasts and inflammatory cells.

### Reduced commensal microbiota exacerbates osteonecrosis induced by periapical inflammation

**Fig. 4:**
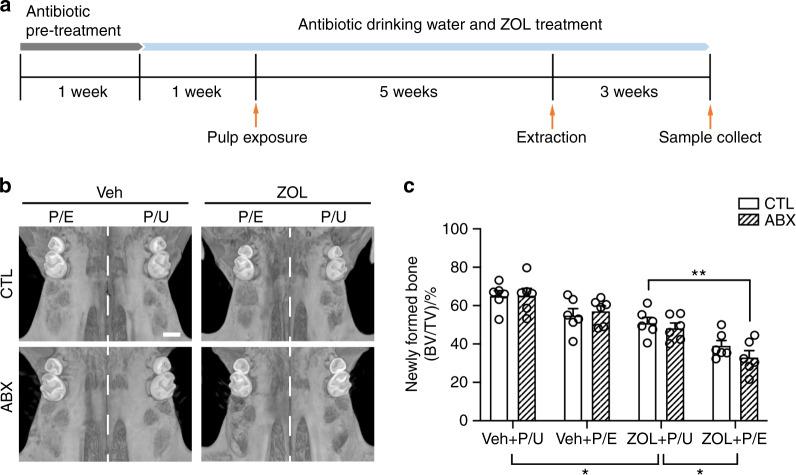
Establishment of pulp exposure and tooth extraction MRONJ mice model with broad-spectrum antibiotic treatment. **a** Schematic diagram of the experiment. For a detailed description, please refer to section of “Materials and methods”. **b** The uCT scans of the maxillae after the sample were harvested. **c** Quantification of newly formed bone in tooth extracted socket (BV/TV). P/E pulp exposed, P/U pulp unexposed. All quantified data represent mean ± SEM (*n* = 6). *P < 0.05; **P < 0.01. Scale bar: 500 μm

To determine the role of the commensal microbiota in the onset of ONJ development, we implemented our previously established MRONJ mouse model. The left side of the maxillary first molar was exposed to induce periapical periodontitis and after 5 weeks the extraction were then performed on the same tooth as well as the contralateral first molar. Then after 3 weeks of healing, the samples are harvested (Fig. 4a). Consistent with our previous study, µCT images showed that unfilled sockets were present in pulp-exposed tooth-extracted sites of zoledronic acid (ZOL)-treated group,^27^ and they were more prominent in ABX-treated group (Fig. 4b). To determine whether the bony sequelae in the tooth-extracted socket is associated with pulp-exposure induced ONJ under ABX treatment, we quantify the newly formed bone in tooth extracted socket. ZOL treatment can overall reduces new bone formation, indicating the inductive role of ZOL in tooth extraction-induced ONJ development through inhibiting or delaying the healing process. With pulp exposure, ABX treatment further inhibited new bone formation compared with control mice in ZOL-treated group (Fig. 4c), which suggests that the depletion of microbiota might cause more severe damage to the alveolar bone with the ONJ development.

**Fig. 5:**
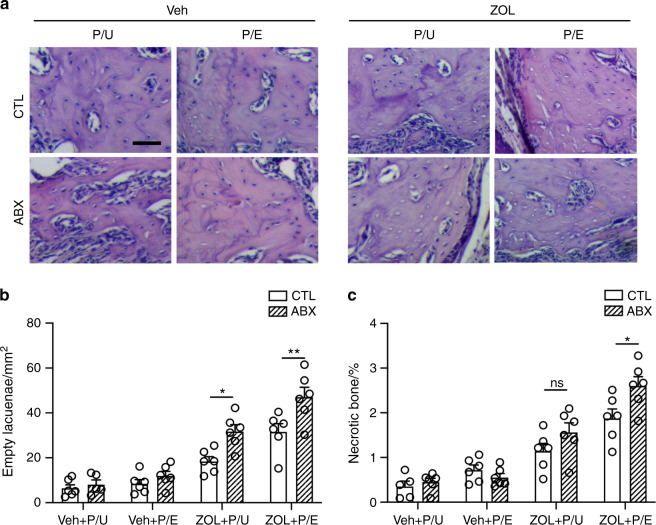
Reduction of commensal microbiota exacerbates periapical periodontitis-mediated MRONJ development. **a** Representative images of H&E staining at the site of extraction. **b** Quantification of empty lacunae number. **c** Quantification of necrotic bone presented as a percentage of the total bone area. P/E pulp exposed, P/U pulp unexposed. All quantified data represent mean ± SEM (*n* = 5–6). *P < 0.05; **P < 0.01; ns, not statistical significant. Scale bar: 50 μm

**Fig. 6:**
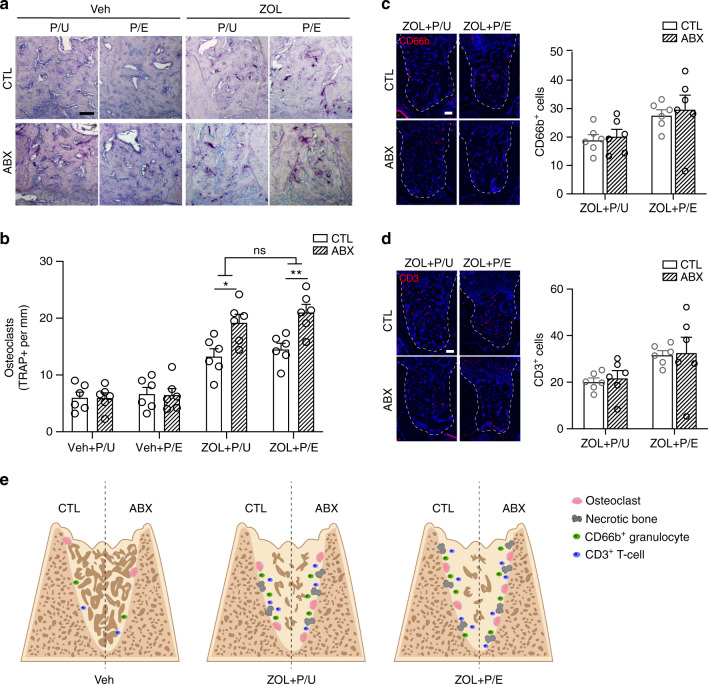
Assessment of osteoclasts and immune cells in dysbiotic mice. **a** TRAP staining with aniline blue counterstain at the tooth extracted site. **b** Quantification of TRAP + osteoclast number per bone surface. **c**, **d** Representative images and quantification of immunofluorescent staining for CD66b^+^ (**c**) and CD3^+^ (**d**) cells at the tooth extracted site in BP treated mice. Dashed lines outline the area where CD66b^+^ and CD3^+^ cells were counted. **e** Proposed model with tooth extraction. Antibiotic (ABX) does not alter the number of osteoclasts or necrotic bone formation in Veh mice (left). Under zoledronic acid (ZOL) treatment (center and right), ABX treatment leads to increased necrotic bone and osteoclast number. ABX does not alter the number of CD66b^+^ and CD3^+^ immune cells regardless of pulp exposure. P/E pulp exposed, P/U pulp unexposed. All quantified data represent mean ± SEM (*n* = 6). *P < 0.05; **P < 0.01; ns, not statistical significant. Scale bar: 100 μm

Osteonecrosis was then evaluated the by the number of empty lacunae and amount of necrotic bone through histological approach. The result showed that with the extraction, the presence of BPs induced empty lacunae and necrotic bone, and pulp exposure could further induce osteonecrosis in ZOL-treatment group (Fig. 5a–c, *P* < 0.05). Interestingly, ABX treatment exacerbated osteonecrosis demonstrated by significantly increased necrotic bone and empty lacunae in pulp-exposed site with ZOL administration (Fig. 5a–c, *P* < 0.05). Further analysis showed that more TRAP + osteoclasts were exhibited in ZOL-treated mice regardless of pulp exposure (Fig. 6a, b, *P* < 0.05). Within the ZOL-treated group, ABX administration could induce the number of TRAP + osteoclasts, which is more obvious in the pulp-exposed site (Fig. 6a, b, *P* < 0.05). When we examined CD66b^+^ and CD3^+^ cells, we found no difference in their numbers regardless of antibiotic treatment (Fig. 6c, d), suggesting persistent ongoing inflammatory responses. Taken together, our results indicate that with BPs and pre-existing periapical disease, ABX treatment induces osteonecrosis formation and osteoclasts number, but does not significantly affect the number of CD66b^+^ granulocytes and CD3^+^ T-cells (Fig. 6e).

## Discussions

Bacterial infection is a common finding in MRONJ patients and the degree of infection is associated with MRONJ advancement.^23,29^ Indeed, the use of antibiotics has been suggested as a standard of care in patients with ongoing MRONJ.^3^ However, the etiological role of bacterial infection that may cause MRONJ development in the beginning stage is not clearly defined.

Depletion of indigenous commensal oral and GI microbiota using antibiotic treatment is routinely used to examine the role of commensal microbiota.^30^ Previously, we used the similar method to reduce the indigenous microbiota in mice that are pre-disposed to inflammatory condition (e.g., ligature-induced periodontitis) and subsequent tooth extraction in the presence of bisphosphonate.^25^ LIP is a well-established model to mimic periodontitis in mice because ligature entraps bacteria and accumulation of dental plaque.^31,32^ However, the ligature placement also causes the physical trauma, complicating interpretation whether inflammation is truly induced by bacteria or consistent physical trauma by ligature itself. In this study, we predisposed the mice to another inflammatory condition (e.g., pulp exposure) and examine the direct effects of reduced commensal microbiota in ONJ development. The current study demonstrated that commensal microbiota did not contribute to the etiological role of initiating MRONJ development; rather, significant reduction of commensal microbiota exacerbated development of MRONJ (Fig. 5a–c), suggesting that indigenous microbiota may have protective role in ONJ development and that a delicate balance between reducing pathologic bacteria and preserving the indigenous oral flora is an important factor in ONJ development.

Previously, it was shown that BPs disturbed the healing process by inhibiting woven bone formation in the tooth-extracted sockets.^27,33^ Consistent with this observation, the current model also caused diminished new bone formation in the tooth-extracted sites (Fig. 4b, c). BPs inhibit appositional new bone formation by impairing osteoclasts’ resorptive function and osteoclast–osteoblast coupling.^33,34^ Interestingly, germ-free condition in mice is associated with increased bone mass and diminished osteoclast differentiation.^35^ Given that BPs are known to increase the numbers of non-attached osteoclasts,^36^ and that both BPs and ABX impair osteoclasts’ functions, it explains increased bone necrosis (Fig. 5a–c) and decreased new bone formation in the tooth-extracted sockets (Fig. 4b, c) when there are more osteoclasts present in ZOL and ABX group (Fig. 6a, b).

We previously demonstrated that the use of ABX in mice significantly altered oral microbiota composition in mice by directly measuring 16S rDNA gene copies from bacteria in the ligature as well as examining cecum size and bone volume.^25^ In this study, we were not able to demonstrate directly the composition of the oral microbiota due to technical difficulties in collecting samples from the root canal or periapical lesions. However, enlarged ceca were similarly exhibited in mice with antibiotics (ABX) treatment for four weeks at which the size of the cecum the ABX-treated mice became an average of four times more than that from the CTL mice (Fig. 1b). In addition, ABX treatment significantly increased the bone volume as indicated by the BV/TV ratio (Fig. 1c), indicating that ABX regimen in this study has functionally altered oral microbiota composition in the root canals.

It is noteworthy that, although ABX treatment alone reduced inflammation at the root apex after pulp exposure (Fig. 3b, c), ABX treatment in the presence of ZOL failed to reduce inflammation (Fig. 6c, d). Indeed, while both CD66b^+^ granulocytes and CD3^+^ T-cells, the predominant cells in the innate and the adaptive immune system,^37^ respectively, were decreased by ABX treatment alone, no inflammation was reduced even with ABX treatment in the presence of ZOL. It was previously showed that bisphosphonate-treated osteoclasts triggered increased release of pro-inflammatory mediators such as IL6, TNF-a, and IL-1ß.^38^ In our study, ZOL treatment increased the numbers of osteoclasts (Fig. 6a, b), which is in line with the previous report that dysregulated osteoclasts are known to be associated with long-term bisphosphonate users.^39^ Because bounded form of BPs is highly inert whereas free form of BPs asserts biological activities, it is tempting to speculate that short life span of matured osteoclasts due to free form of BPs while resorbing the bone surface induces increases in osteoclasts formation and homing as a feedback mechanism. As such, it is probable that continual ZOL treatment over long period of time may have caused increased inflammatory condition by recruited osteoclasts that released pro-inflammatory factors.

On the other hand, other agents such as denosumab or bevacizumab that are known to cause ONJ do not induce, if not reduce, inflammation.^40,41^ Although the direct effects of denosumab or bevacizumab on inflammation in the context of MRONJ development warrant further examination, induction of inflammatory signals by the drug itself may not fully explain the persistent inflammation even with the ABX treatment.

Alternatively, persistent inflammation during the ABX treatment may be associated with impaction of debris in the root canals from the oral environment such as food, which was frequently observed in our model (Fig. 2c and 3a). Pulp exposure in germ-free condition also induced small degree of inflammation due to impacted food or debris.^42^ Furthermore, occlusal trauma is also known to elicit pulpal inflammation and exacerbate periapical lesion.^43^ These non-bacteria associated factors may explain persistent pulpal inflammation and continual destruction of the local environment to cause ONJ development in combination with BP.

A previous study showed that the use of antibiotic prophylaxis that starts before 3 days and lasts until 4 days after tooth extraction significantly lowered MRONJ development in rats.^17^ In line with this, ectopic introduction of *Fusobacterium nucleatum* after tooth extraction caused development of MRONJ-like lesions in mice.^44^ As such, prophylactic antibiotic treatment has been recommended in the clinic when a tooth extraction is indicated in patients who are taking anti-resorptive agents. On the other hand, in our study, we significantly reduced the bacterial loads throughout study and found that long-term antibiotic treatment may exacerbate MRONJ development. Based on these notions, it implies that while short-term antibiotic regimen may help reducing the incidence of developing ONJ lesions, a long-term excessive treatment with antibiotics may contribute significantly to ONJ development. Knowing this fine balance of using antibiotic regimens in ONJ patients would be imperative to mitigate this detrimental lesion in the oral cavity.

## Materials and methods

### Animals

Six-week-old C57BL/6J female mice were purchased from the Jackson Laboratories (Stock no. 000664) and kept in a specific pathogen free environment in the UCLA Division of Laboratory and Animal Medicine (DLAM). All experimental protocols were approved by institutional guidelines from the Chancellor’s Animal Research Committee (2011–062).

### The mouse model of periapical lesion

Mice were intraperitoneally injected with ketamine/xylazine (100 and 5 mg kg^−1^ body weight, respectively) for anesthesia. The pulp of left maxillary first molar was exposed by a high-speed 1/4 round bur on a portable dental unit (Aseptico Inc., Woodinville, WA). The pulp exposure was performed under endodontic microscope (BM-LED stereo microscope, MEIJI Techno, Japan) with ×10 magnification. Exposed tooth was left open without any coverage. As a control, contralateral maxillary first molar was not pulp-exposed during the study.

### Commensal microbiota reduction and osteonecrosis development

Mice were assigned into four groups randomly: control (CTL)/Veh, antibiotic-treated (ABX)/Veh, CTL/Zoledronic acid (ZOL), and ABX/ZOL (*n* = 8–10 mice each). During the first 7 days of the study, a mixture of intragastric and intraoral gavage of neomycin (100 mg·kg^−1^), vancomycin (50 mg·kg^−1^) and metronidazole (100 mg·kg^−1^) were administered to mice in ABX/Veh and ABX/ZOL group twice daily while ampicillin (0.5 mg·mL^−1^) dissolved in the drinking water ad libitum. At the same time, the mice in CTL/Veh and CTL/ZOL received sterile saline gavage and unaltered drinking water. After the first 7 days, ABX mice were provided with a mixture of neomycin (1 mg·mL^−1^), vancomycin (0.5 mg·mL^−1^), and ampicillin (1 mg·mL^−1^) in drinking water ad libitum, while 0.9% NaCl or 125 μg·kg^−1^ Zoledronic acid (Sagent Pharmaceuticals) were respectively administered in the Veh and ZOL groups through intravenous injections biweekly for the remainder of the study. One week after the end of gavage administration, pulp of the left maxillary first molar was exposed in all mice to induce periapical periodontitis. The mice that were used to examine periapical periodontitis development were sacrificed 3 weeks after pulp exposure (Fig. 1a). To induce MRONJ in the remaining mice, the exposed tooth as well as the contralateral healthy tooth were extracted after 5 weeks of pulp exposure. After three additional weeks healing, the animals were euthanized by CO_2_ followed by cervical dislocation. Maxilla, femur, and ceca of each mouse were harvested for further analysis. A schematic illustration indicating the timeline of the study is provided in Fig. 3a.

### Micro-computed tomography (μCT)

The mouse maxillae and femur were scanned with a voxel size of 10 μm^3^ through a 1.0 mm aluminum filter at 60 kVp and 166 μA (SkyScan 1275; Bruker). Two-dimensional reconstruction images were generated by N Recon (Bruker) with X-Y alignment and dynamic range adjustment. Three-dimensional representative images were generated in CTVox (Bruker). Morphological parameters of trabecular bone microarchitecture in femur were evaluated by CTAn (Bruker microCT, Kontich, Belgium).^45^ Epiphyseal growth plate was defined to be the starting point of region of interest (ROI) and three-tenths of total femur length was calculated as the size of ROI. Bone volume fraction (BV/TV; %) of the femur from each mouse was collected by measure bone volume (BV; mm^3^) and the total volume (TV; mm^3^). Newly formed bone in the tooth-extracted sites, displayed as percentage of BV/TV, was also quantified by selecting a volume containing the distal lingual, distal buccal, and mesial roots.

### Tissue preparation

The maxillae were harvested and fixed in 4% paraformaldehyde in PBS (pH 7.4) overnight at 4 °C. Then 70% ethanol was used to store these fixed maxillae, which were subjected to μCT scanning. After scanning, the tissue was transferred into daily-changed decalcification solution of 5% EDTA and 4% sucrose in PBS, pH 7.4 at 4 °C for 2–3 weeks, and decalcification solution was changed daily. Decalcified tissues were subsequently sent to UCLA Translational Procurement Core Laboratory (TPCL) for processing and paraffin embedding. The embedded tissue were sectioned at the coronal plane in a series of 5 µm thickness.

### Tartrate-resistant acid phosphatase (TRAP) histochemical staining

TRAP staining were performed following the protocol described previously.^33^ Four slides per sample were deparaffinized at 60 °C, then rehydrated in ethanol with an increasing concentration of water. The rehydrated tissue slides were stained with TRAP solution (#387A-1KT, Millipore Sigma) for 30 min in dark, then washed with water and counterstained with hematoxylin for 8 s. The number of osteoclasts which were identified with the presence of multiple nuclei (*n* > 5) was counted using ImageJ software after the pictures were taken by microscope (model DP72; Olympus) at ×100 magnification.

### Histomorphometric analysis

Empty lacunae number and necrotic bone areas were measured according to previously described protocol.^33^ H&E staining was performed to the sectioned slides (*n* = 4, every five cuts) from each sample. After the tissue were rehydrated, the sectioned slides were stained with hematoxylin for 2.5 min, and then washed with water and 95% ethanol before stained with eosin for 1 min. The stained slides were then dehydrated in 70%, 95%, and 100% ethanol followed by xylene. Slides were mounted using mounting medium (Permount; Fisher Scientific, Houston, TX) and digital images were taken by microscope (model DP72; Olympus) at ×100 magnification. The total bone surface area was measured using ImageJ software, and the number of empty lacunae per total bone area (#/mm^2^) were calculated. Necrotic bone was determined as a bone area that has at least five empty lacunae per 1 mm^2^, and the total necrotic bone areas will be divided by total bone area to get the percentage of necrotic bone.

### Immunofluorescence staining

Paraffin embedded tissues were rehydrated, and antigen retrieval was performed in citrate buffer at 60 °C overnight. Sections were blocked in 10% normal goat serum and incubated in primary antibody against CD3 (1:200, #ab5690, Abcam) and CD66b (1:200, #ab197678, Abcam) diluted in 3% serum overnight. After washes in PBST for three times, incubation with secondary antibody diluted in 3% serum was performed for 1 h followed by counterstaining with DAPI for 8 min. Slides were mounted using Prolong Gold (P36930; Thermofisher Scientific) and images were taking using a confocal microscope (LSM700; Zeiss). CD3^+^ and CD66b^+^ cells were counted in the area (outlined by dashed lines in Figs. 3b, c and 6c, d) around the root apex after pulp exposure or within the tooth extracted socket after tooth extraction.

### Statistical analysis

Single factor ANOVA and Tukey’s post hoc test were performed to compare the μCT data of trabecular bone measurements (BV/TV), empty lacunae number, necrotic bone (%), and number of osteoclast among different groups. All statistical analyses were performed through Prism 8 software.

## Data Availability

All data used/analyzed in this article are contained within the manuscript or available from the corresponding authors upon reasonable request.
